# Oral Microbiome Signatures in Hematological Cancers Reveal Predominance of *Actinomyces* and *Rothia* Species

**DOI:** 10.3390/jcm9124068

**Published:** 2020-12-17

**Authors:** Jean-Luc C. Mougeot, Micaela F. Beckman, Holden C. Langdon, Michael T. Brennan, Farah Bahrani Mougeot

**Affiliations:** Carolinas Medical Center-Atrium Health, 1000 Blythe Blvd, Charlotte, NC 28203, USA; micaela.beckman@atriumhealth.org (M.F.B.); holden.langdon@atriumhealth.org (H.C.L.); mike.brennan@atriumhealth.org (M.T.B.)

**Keywords:** oral microbiome, hematological or blood cancers, next-generation 16S sequencing, LEfSe biomarkers, *Actinomyces*

## Abstract

The endogenous microbiome of healthy individuals in oral cavities is diverse, representing over 700 bacterial species. Imbalance in oral and gut microbiome composition and associated gene expression has been linked to different forms of hematological (blood) cancers. Our objective is to compare oral microbiome profiles of patients with blood cancers (BC group: N = 39 patients, n = 124 oral samples) to those of healthy control subjects (HC group: N = 27 subjects, n = 100 oral samples). Saliva samples and swabs of buccal mucosa, supragingival plaque, and tongue were collected from blood cancer patients and healthy controls. Next-generation sequencing (16S-rRNA gene V3–V4 region) was used to determine the relative abundance of bacterial taxa present at the genus and species levels. Differences in oral microbiome *beta*-diversity were determined using multivariate permutational analysis of variance (PERMANOVA). Linear discriminant analysis (LDA) effect size (LEfSe) analysis was performed to identify differentiating bacterial taxa in pairwise comparisons. The PATRIC_v3.6.7_ online tool was used to determine the predominance of potential pathogenicity in the BC group. The oral microbiome *beta*-diversities of the BC and HC groups differed and corresponded to a reduced *alpha*-diversity in the BC group. LEfSe analysis showed significant LDA scores for *Actinomyces* and *Rothia* spp., differentiating the BC group from the HC group. In silico analysis using PATRIC_v3.6.7_ demonstrated that the groups of bacteria possessing traits of “antibiotic resistance”, “oral pathogen”, and “virulence” was enriched in the BC group. Although 56% of the BC patients received antibiotics within two weeks of the oral bacterial sampling, *Actinomyces genus* remained the top differentiating feature in the BC group regardless of the administration of antibiotics, while *Rothia dentocariosa* was detected as the top differentiating feature in the BC patients who did not receive antibiotics, but not in those who received antibiotics. Further investigation is needed to better understand the interactions of certain oral species with the host immune system to better characterize clinically relevant associations with hematological cancers.

## 1. Introduction

A recent review by Siegel et al. reported that in 2020, there will be approximately 180,000 new cases of hematological/blood cancers (i.e., Hodgkin and non-Hodgkin lymphomas, myeloma, and leukemia) and about one-third will succumb to the disease [1]. In spite of significant advancements in diagnostics and treatment protocols for cancer patients [2,3], there is still a gap in knowledge to understand the underlying factors contributing to the progression of many cancers. This is in part due to insufficient knowledge about the effects of the human microbiome on carcinogenesis and on host immune response [4].

Historically, most research has focused on the determination of associations between the oral and/or gut microbiome with solid-tumor cancers. Mohammed et al. [5] showed a significant increase in 31 bacterial species in saliva samples of pancreatic cancer patients, including *Porphyromonas gingivalis* and *Aggregatibacter actinomycetemcomitans*, the species known to be associated with tooth decay and infective endocarditis [6]. Additionally, an elevation in the blood serum antibodies against *P. gingivalis* has been suggested to be associated with an increased risk of pancreatic cancer and liver cirrhosis [5]. In another study of pancreatic cancer, an increase in the abundance of a *Fusobacterium* spp. in pancreatic cancer tissues was found to be associated with a worse prognosis [7]. *Fusobacterium* has also been identified as an indicator of colorectal carcinogenesis [8]. *F*. *nucleatum* has been found to be present in the stool samples and tumor biopsies of colorectal cancer patients [8]. Indeed, this species has been used in Japan as the biomarker for screening patients to determine their prognosis [9]. Furthermore, an increase in the relative abundance of *Fusobacterium*, *Haemophilus*, *Neisseria*, and *Proteobacteria* and a decrease in the relative abundance of *Porphyromonas* on the tongue have been associated with gastric cancer [10]. A possible link between the oral microbiome and carcinogenesis has also been shown in esophageal cancer [11]. Chen et al. [11] found an increase in *Prevotella, Streptococcus,* and *Porphyromonas* genera in saliva samples of patients with esophageal squamous cell carcinomas compared to healthy controls. The authors also identified the pathway through which *Streptococcus* contributes to carcinogenesis of esophageal tissue through the initiation of inflammation and development of dysplasia [11].

Unlike solid tumor cancers, there is little known about the association of the oral microbiome with the various types of hematological cancers. Distinct gut and oral (dental plaque) microbiome profiles associated with acute lymphoblastic leukemia (ALL) were identified in the pediatric population [12,13,14]. In this population, a reduction in gut microbial diversity, along with an increased abundance of phylum Actinobacteria, was associated with increased immune activation in ALL survivors who developed chronic inflammation [12]. In another study, Galloway-Pena JR et al. showed an unstable temporal microbial diversity in the gut and oral cavity of patients with acute myelogenous leukemia (AML) [15]. Furthermore, a study of patients undergoing hematopoietic stem cell transplants showed oral microbiome changes associated with respiratory signs and symptoms [16]. Additionally, we recently reported that lasting changes in oral *Gammaproteobacteria* profiles occur in hematopoietic stem cell transplant patients who develop ulcerative oral mucositis after conditioning therapy [17].

In the present study, we determine the differences between oral microbiome profiles of patients with hematological cancers of various types and healthy controls, based on multiple oral site sampling and next-generation sequencing of the 16S rRNA bacterial gene (V3–V4 region). 

## 2. Materials and Methods

### 2.1. Patient Recruitment 

Patients (pts; N = 39) diagnosed with hematological/blood cancers (BCs), who were scheduled for conditioning treatment prior to hematopoietic stem cell transplant, were recruited at Atrium Health’s Carolinas Medical Center, Charlotte, North Carolina, with the approval of the institutional review board. Additionally, healthy control subjects (HC; N = 27) were recruited for this study. All participants gave informed consent for the study. Hematological cancer diagnoses included acute lymphoblastic leukemia (ALL, N = 3), acute myelogenous leukemia (AML, N = 16), chronic myelogenous leukemia (CML, N = 1), lymphoma (LYM, N = 10), myelodysplastic syndrome (MDS, N = 2), myelofibrosis (MF, N = 2), and multiple myeloma (MM, N = 5). Samples were collected before conditioning therapy at baseline. Of the 39 hematological cancer patients, 22 were treated with antibiotics within two weeks before sample collection. None of the healthy controls were treated with antibiotics within this time frame. Demographics and clinical characteristics of BC pts, HCs, and the largest cancer subgroup, AML pts, are presented in Table 1. Although no significant difference was found for age and gender, black ethnicity was over-represented in the BC group of patients.

### 2.2. Sample Collection, Bacterial DNA Extraction and Sequencing

Oral samples, i.e., buccal mucosa (B), superficial supragingival plaque (P) and tongue (T) swabs, and saliva sample (S), were collected from BC pts at baseline (precancer treatment) and HCs.

Saliva collection was performed first while chewing unsweetened and unflavored gum (The Wrigley Company, Mars, Inc., Chicago, IL, USA) for a period of two minutes into a 50-mL conical BD Falcon^TM^ polypropylene centrifuge tube (Corning, NY, USA) and kept on ice for no longer than 30 min before processing or being stored at −80 °C. Buccal mucosa samples were subsequently collected by swabbing both sides of the buccal mucosa for 10 s each. Tongue samples were then obtained by swabbing a 1 ${\mathrm{cm}}^{2}$ region on both sides of the middorsal region of the tongue for 5 s. Finally, superficial supragingival plaque (P) samples were obtained using OmniSwabs (GE Life Sciences-Buckinghamshire, UK) across the lateral surfaces of all maxillary and mandibular teeth at the junction of each tooth and gingiva.

Bacterial genomic DNA was extracted from oral samples using the QIAamp DNA Mini Kit procedure (QIAGEN, Valencia, CA, USA) per the manufacturer’s instructions. Identification of bacterial genera and species was performed by utilizing Human Oral Microbe Identification using *Next Generation Sequencing* (HOMI*NGS*), which employs a ProbeSeq BLAST-type program for species/genera identification through recognition of the 16S rRNA gene (V3–V4 region). During sample preparation, 50 ng of genomic DNA was used for PCR, in which the 16S rRNA (V3–V4) region was amplified, followed by purification and processing methods described by Caporaso et al. using MiSeq (Illumina, Inc., San Diego, CA, USA) [18]. ProbeSeq sequence identification used rRNA-based in silico probes in a BLAST program to determine the species/genera counts. The sequence-reads were matched to 737 ProbeSeq taxon probes, i.e., to a species probe (n = 620) or a genus probe (n = 117) if not matched to a species probe, or were otherwise recorded as an unmatched read.

### 2.3. Data Preprocessing

Results were provided as Excel spreadsheets displaying total probe counts (matched reads) per taxon per patient ranging from 0–89,766 for species probes and 0–133,658 for genus probes (BC pts and HC data combined). Probe counts were summed for the species and genera identified through several probes, resulting in a dataset allowing the identification of 534 species and 78 genera from the initial 620 species and 117 genus probes, respectively. Probe counts per species or genus were converted into relative abundances, based on the total number of counts per patient for all the species and genus probes combined. HC subjects and BC pts were categorized per oral sample site combinations to perform PERMANOVA. The combinations BPST, BST, PST, and ST yielded the largest subcohort sizes associated with the largest number of successfully processed oral samples (spl.), i.e., BPST (20 HC/80 spl.; 13 BC/52 spl.), BST (24 HC/72 spl.; 17 BC/51 spl.), PST (23 HC/69 spl.; 29 BC/87 spl.), and ST (27 HC/54 spl.; 39 BC/78 spl.). The combinations BPT and BPS yielded 20 HC/13 BC and 19 HC/13 BC, respectively, with all BC samples stemming from the BPST cohort, and, therefore, were not considered for further analysis.

Overall, for the samples analyzed, there was no significant difference regarding unmatched reads between the HC group (range 4.00% to 49.20%, mean (SD) = 19.20 (10.37)) and the BC group (range 2.77% to 45.15%, mean (SD) = 20.37 (10.32)) (*p* > 0.05, Mann–Whitney U-test). In addition, there was no inverse correlation between the percentages of unmatched reads and the number of species and genera detected for both the HC and BC groups (Spearman’s r = +0.30772 and +0.3736, respectively, *p* < 0.05). These data indicate that an increase in unmatched reads did not result in a loss of detection of species due to the generation of spurious sequences (i.e., caused by lower bacterial DNA quality).

### 2.4. Statistical Analysis

#### 2.4.1. PERMANOVA

PRIMER_v7_ software (PRIMER-E Ltd., Ivybridge, UK) was used to run a multivariate permutational analysis of variance (PERMANOVA) using unrestricted permutation of raw data (one factor) or permutation of residuals under a reduced model (2–3 factors), 9999 permutations, and type III partial sum of squares. Two types of analyses (AN) were performed: (i) blood cancer (BC) vs. healthy controls (HC) (AN1) and (ii) a subanalysis for the blood cancer type AML, which had the largest cancer subgroup sample size (AN2), to compare *alpha*- and *beta*-diversities based on relative abundance data and different sample site combinations. For the sample site combinations BPST, BST, PST, and ST, BC pts were compared to HCs within the limit of available data, i.e., N = 39 pts, n = 124 oral samples for the BC group and N = 27 pts, n = 100 oral samples for the HC group (AN1; Table 1). In AN2, AML subgroup (N = 16 pts, n = 49 samples) was compared to the HC group.

The PERMANOVA design consisted of the fixed variables “Group” (BC, HC) and “Site” for the abovementioned sample site combinations. To increase the degrees of freedom, the design also combined the random variables “antibiotics” (Yes/No) and “diagnosis” (HC, ALL, AML, CML, LYM, MDS, MF, MM) into a single variable designated as “AB-DIA” (i.e., coded as YAML or NMM, for example), which was nested into “Group” and “Site” in a three-factor PERMANOVA design. Indeed, AB treatment frequency was dependent on the type of blood cancer (ALL 2/3, AML 11/16, CML 0/1, LYM 6/10, MDS 1/2, MF 2/2, and MM 0/5), while the absence of AB treatment characterized the HC group. Secondary variables such as age, gender, and ethnicity, potentially affecting microbiome profiles, were not included in the analyses due to sample-size limitations.

Relative abundance data were squared-root transformed and converted to Bray–Curtis similarity matrices prior to PERMANOVA analysis. For all analyses performed, PERMANOVA Monte-Carlo-corrected *p*-values (*p* < 0.05) were obtained for both fixed and random variables.

#### 2.4.2. Principal Coordinate Analysis (PCoA) and Nonmetric Multidimensional Scaling (nMDS)

For the selected comparisons, PST AN1, and AN2 that were statistically significant per PERMANOVA, nonmetric multidimensional scaling PCoA, and nMDS plots of BC and HC samples were generated using PRIMER_v7_ software (PRIMER-E Ltd.).

#### 2.4.3. Linear Discriminant Analysis (LDA) Effect Size (LEfSe)

LEFSe was performed on BPST, PST, and ST cumulative relative abundance data in AN1 and PST in AN2 using the online tool Galaxy_v1.2_ [19] in order to determine differentiating features at the genus and species levels, as described by Segata et al. [20]. Taxonomy levels were manually added to all groupings within the text files that were formatted according to Galaxy_v1.2_ required formatting. Data formatting and input consisted of diagnosis (blood cancers and healthy control) as class and patient ID as subject. Analysis strategy “one-against-all” was used for multiclass analysis; the factorial Kruskal-Wallis test, as well as pairwise Wilcoxon signed-rank test for all groupings, were set at a Monte-Carlo (*alpha* = 0.05). Results were displayed as histograms and cladograms, representing taxa with an LDA > 3.0 threshold for the analyses AN1 BC vs. HC, AN1 BC—No Antibiotics vs. HC, AN1 BC—Antibiotics vs. HC, and AN2 AML vs. HC (AML subgroup, with or without the inclusion of antibiotic treatment).

#### 2.4.4. *Alpha*-Diversity

The Shannon *alpha*-diversity index was determined using PRIMER_v7_ software for the AN1 and AN2 comparisons, based on relative abundance derived from cumulated counts for BPST and PST site combinations. Significance was determined by Mann–Whitney U-test (*alpha* = 0.05).

#### 2.4.5. Association between Taxonomic Profiles and Pathogenicity

The PATRIC_v3.6.7_ online tool [21] was used to link the taxonomic profiles to pathogenicity concepts (i.e., oral pathogen, virulence, and antibiotic resistance) for the AN1 and AN2 analyses based on LEfSe significant species and genera. Cumulative mean relative abundance data plots for species representing enriched pathogenicity concepts were generated using the PST site combination (i.e., representing the largest number of patients and associated oral samples).

### 2.5. Ethics

This study has been approved by the Institutional Review Board of Atrium Health, Charlotte, NC, USA (IRB# 05-14-18B). All patients participating in this study gave informed consent.

## 3. Results 

**Figure 1:**
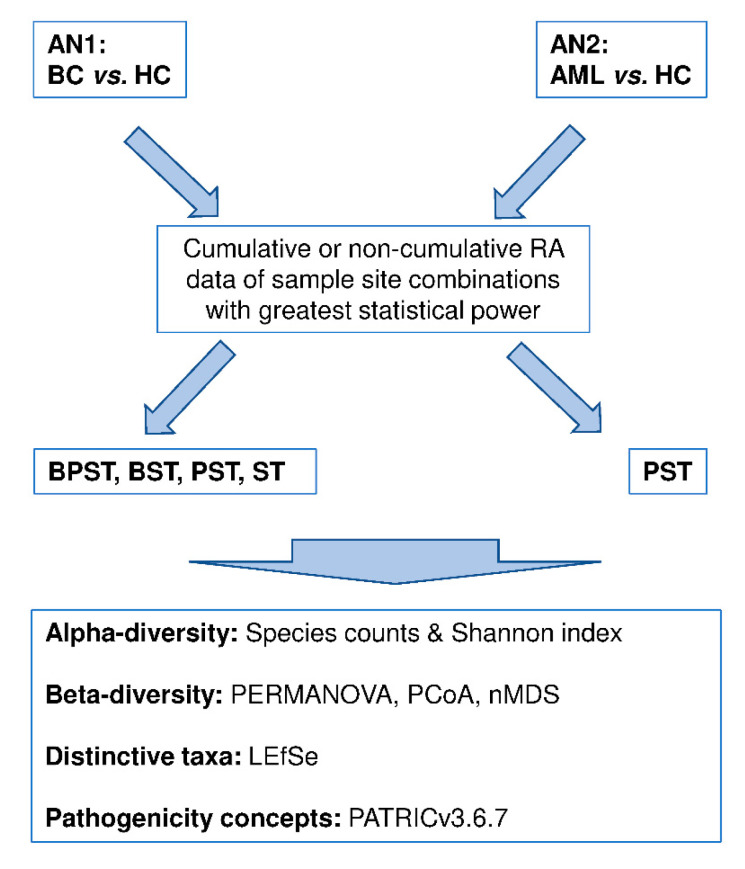
Analytical design to assess differences in oral microbiome *alpha-* and *beta*-diversities and distinctive taxon features in patients with hematological cancers. Oral microbiome *alpha*- and/or *beta*-diversity differences were determined through two categories of analyses: BC vs. HC (AN1) and AML (largest BC patient subgroup) vs. HC. Sample site combinations considered for analysis were BPST, BST, PST, and/or ST. For these sample site combinations, abundance data were cumulative or not for conversion into relative abundance (RA). PRIMER_v7_ software, the Galaxy_v1.2_ online tool, and the PATRIC_v3.6.7_ online tool were used to determine bacterial diversity, distinctive features, and enriched pathogenicity. Oral microbiome sample sites were buccal mucosa (B), superficial supragingival plaque (P), saliva (S), and tongue (T). BC is blood cancer; HC is healthy control; AML is acute myelogenous leukemia.

Figure 1 presents the overall analytical strategy.

### 3.1. BC pts Were Found to Have a Reduction in Alpha-Diversity When Compared to HC

Considering patient demographics and clinical characteristics, no significant difference (*alpha* = 0.05) was found for age (Mann–Whitney U-test) or gender (chi-squared test). Black ethnicity was determined to be over-represented in the BC group of patients compared to healthy control subjects (*p* = 0.0046 per chi-squared test). Demographics and clinical characteristics of BC pts, HC, and the largest cancer subgroup, AML pts, are presented in Table 1. For our cohort of hematological/blood cancer patients (BC; N = 39) and healthy control subjects (HC, N = 27), considering the total possible identification of 534 species and 78 genera through the respective species and genus probes, the average number of species and genera detected per subject was determined for all sample site combinations (BPST, PST, BST, and ST). There were 396 species and 60.6 genera detected on average, with an average of 138 species and 17.4 genera not detected with the abovementioned combinations, whether or not abundance data were cumulated for the different sample site combinations (Table S1).

The average number of species and genera identified per subject in each group (HC, BC, and AML) for the different sample site combinations analyzed (with cumulated RA data) is described in Table S2. A significant reduction in the number of species and genera detected in the BC group/AML subgroup was observed compared to HC, regardless of the sample site combinations (Table S2). For the taxa detected in the PST oral site combination, which had the largest number of patients and associated oral samples (23 HC/69 spl.; 29 BC/87 spl.) based on cumulative PST RAs, there was a significant reduction of the number of species detected in BC per patient (mean = 118.48) compared to HC (mean = 172.78) (*p* < 0.00001; Mann–Whitney U-test). The total number of species detected in the cumulative PST combination was 360 in the HC group and 351 in the BC group. The Shannon index was, on average, significantly different between the BC and HC groups, with BC mean H’ (SD) = 2.59 (0.61) and HC mean H’ (SD) = 2.96 (0.41) (*p* = 0.03236; Mann–Whitney U-test). These results confirm the reduction in *alpha*-diversity in the BC group.

### 3.2. Beta-Diversity Shows Significant Difference between the BC and HC Groups

**Figure 2:**
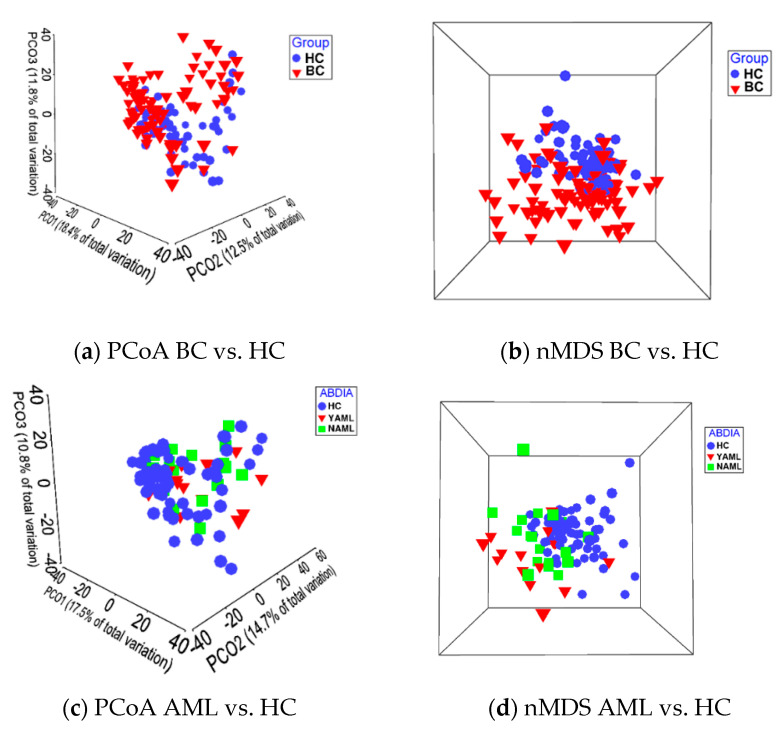
Clustering of oral samples from healthy controls and patients with hematological cancers. Cluster analysis by principal coordinates analysis nonmetric multidimensional scaling was performed in PRIMER_v7_ software using the PST sample site combination. PERMANOVA testing for the PCoA and nMDS comparisons shown in the figure resulted in Monte-Carlo *p* < 0.05. PERMANOVA was based on the initial detection capability of all 737 probes, comprised of 620 species and 117 genus probes. Patients had next-generation sequencing data for saliva sample, buccal mucosa, tongue, and superficial supragingival plaque swabs. (**a**) AN1: BC vs. HC, with a total of three principal coordinates of 30.9% variation; (**c**) AN2: AML vs. HC, with a total of three principal coordinates of 43% variation. (**b**,**d**) 3D nMDs stress value was less than 0.2 for AN1 and AN2. YAML: patients diagnosed with AML that were treated with antibiotics within 2 weeks of sampling; NAML: patients diagnosed with AML that were not treated with antibiotics.

Results from PERMANOVA analyses are presented in Table 2. Noncumulative sample site combinations showed distinct *beta*-diversity between the BC vs. HC groups, regardless of the sample site combination (i.e., BPST, PST, BST, and ST) and antibiotic use. A significant loss of power was observed when reducing the sample sites analyzed for the secondary variables “Site” and “AB-DIA”. In the comparison AML vs. HC, PERMANOVAs were significant, regardless of whether samples from AB-treated or -untreated AML patients were analyzed in combination or separately (Table 2). Moreover, when removing diagnoses with very small sample sizes, there is little effect on the PERMANOVA *p*-value (Table 2). PCoA and nMDS plots for AN1 and AN2 (all AML), showing distinct clustering and significant variation between the BC and HC groups, are illustrated for the PST site combination (noncumulative RA data) in Figure 2.

### 3.3. Actinomyces Genus and Rothia Dentocariosa Account for a Large Difference between the BC and HC Groups

**Figure 3:**
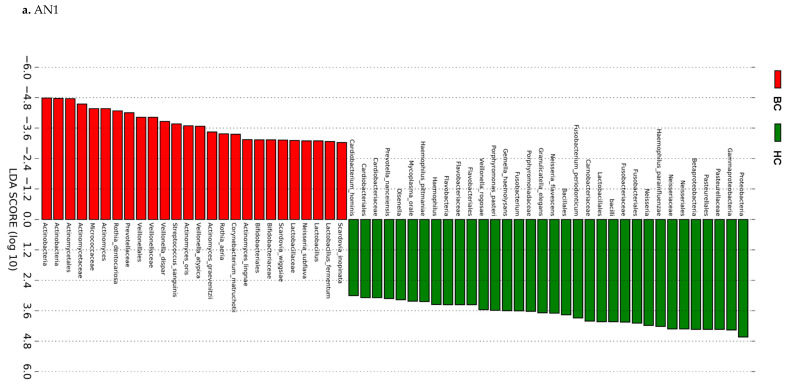

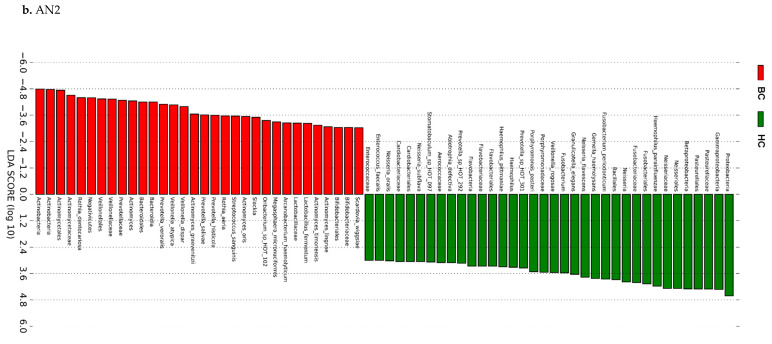

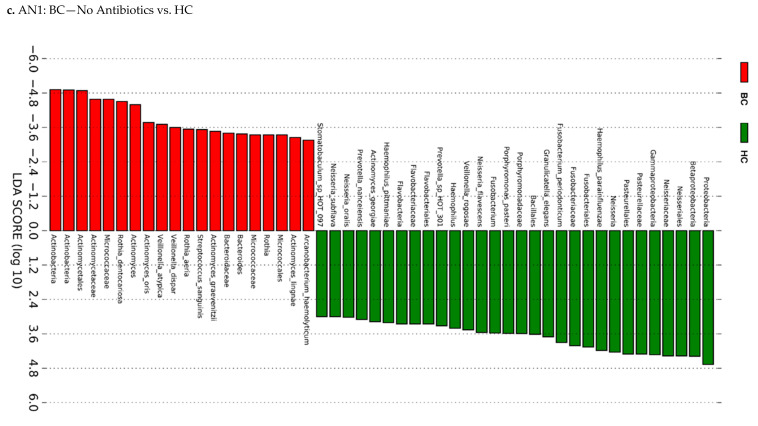

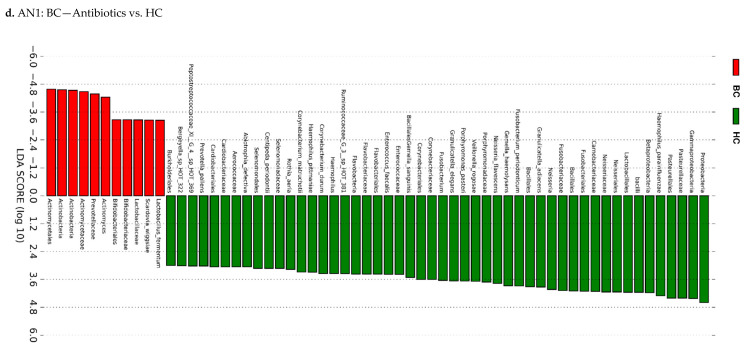

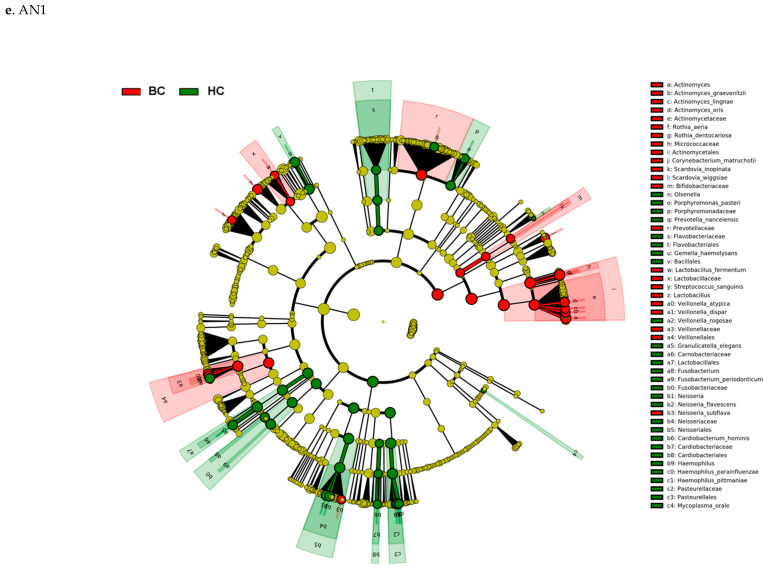

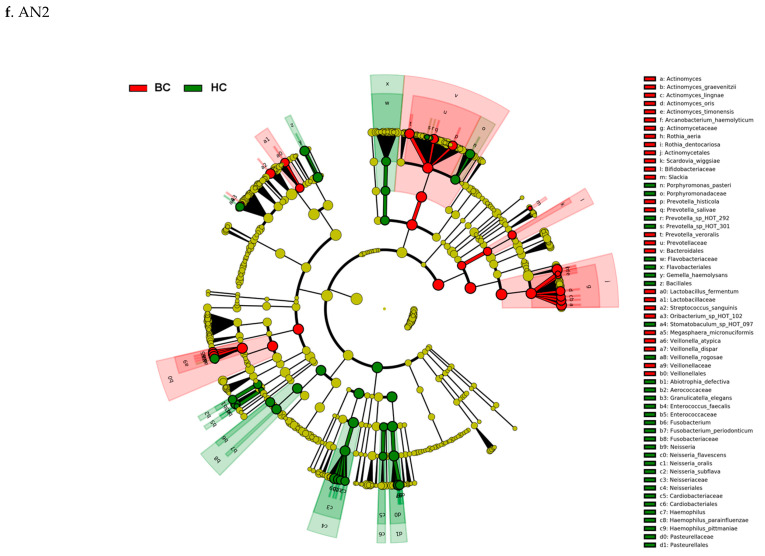

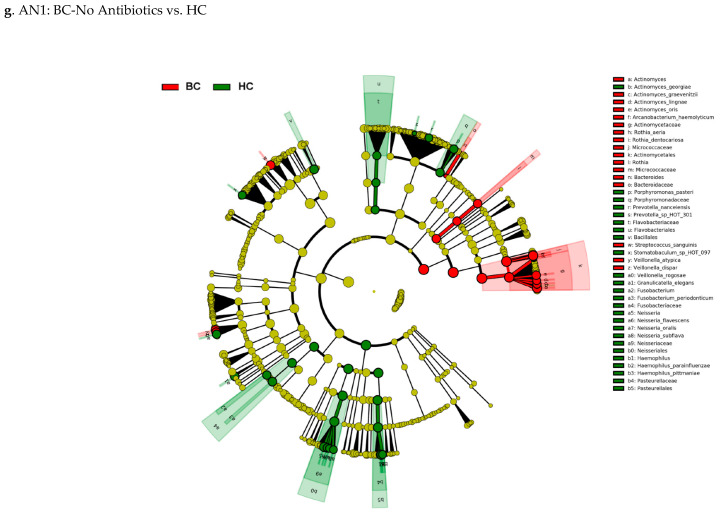

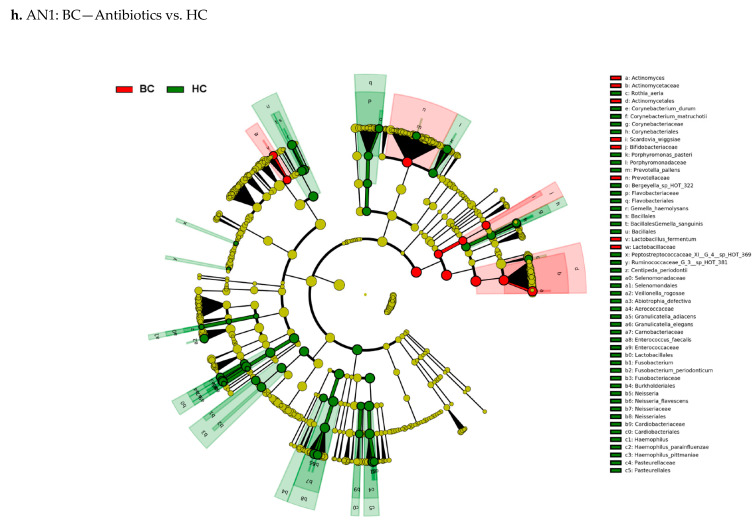
Linear discriminant analysis effect size (LEfSe) histograms and cladograms for the comparisons of hematological cancers (BCs) versus healthy controls (HCs). LEfSe was performed to determine distinct oral microbiome features in samples (saliva sample, buccal mucosa, tongue, and superficial supragingival plaque swabs) from the BC and HC groups. Data formatting and input consisted of diagnosis (BC and HC) as class and patient ID as subject, with an LDA threshold of 3.0. (**a**) AN1: LEfSe results showing a horizontal histogram of hematological cancer patient samples using cumulative PST data. There were 34 HC features and 26 BC features. (**b**) AN2: horizontal histogram comparing differential features of AML patients (red) and HCs (green), representing top 68 differential features using cumulative PST data. (**c**) AN1: BC—No Antibiotics vs. HC horizontal histogram of cumulative PST data where patients with BC were not treated with antibiotics, representing the top 50 differential features. (**d**) AN1: BC—Antibiotics vs. HC horizontal histogram of cumulative PST data where patients with BC were treated with antibiotics, representing the top 61 differential features. (**e**) AN1: LEfSe Cladogram with 60 differential features identified by LEfSe using PST cumulative data. The largest (genus/species level) differentiating features for BC pts were *Actinomyces* genus and *Rothia dentocariosa.* (**f**) AN2: LEfSe cladogram with 68 differential features identified by LEfSe using PST cumulative data. The largest differentiating features for BC pts were *Actinomyces* genus and *Rothia dentocariosa.* (**g**) AN1: BC—No Antibiotics vs. HC LEfSe cladogram, with 50 differential features identified by LEfSe using PST cumulative data, where BC pts were not treated with antibiotics. The largest differentiating (genus/species level) features for BC pts were *Actinomyces* genus and *Rothia dentocariosa*. (**h**) AN1: BC—Antibiotics vs. HC LEfSe cladogram with 61 differential features identified by LEfSe using PST cumulative data, where BC pts were treated with antibiotics. The largest differentiating (genus/species level) features for BC pts were *Actinomyces* and *Scardovia wiggsiae*. Note: genus probe results, representative of family, order, class, and phylum, were included in histograms and cladograms.

Determination of microbial species that may influence the difference in *beta*-diversity among the two groups through LEfSe analysis identified 13 species and 2 genera differentiating hematological cancer patients from healthy controls (AN1) using the PST cumulative sample site combination. The most differential features for all sample site combinations considered for the BC group were *Actinomyces* genus, *Rothia dentocariosa*, and *Veillonella atypica*. LDA scores ranged from −4.8 to 4.8 for the BC and HC groups, respectively (Figure 3a). Further analysis indicated 17 species and 2 genera differentiating AML patients from healthy controls (AN2; Figure 3b). Subanalysis to show the consistency of previous AN1 results, regardless of antibiotic use, showed that *Actinomyces* genus and *Rothia dentocariosa* were top differentiating features for BC pts that did not receive antibiotics (Figure 3c). Additionally, *Actinomyces* genus remained the top genus-level differentiating feature in patients that had received antibiotics (Figure 3d). When diagnoses with very small sample sizes were removed from the PST cumulative sample site combination (leaving AML, LYM, and MM pts), *Actinomyces* remained the top genus-level differentiating feature of BC pts compared to HC. Furthermore, *Actinomyces* and *Rothia dentocariosa* were top features for the PST noncumulative sample site combination when only AML, LYM, and MM were used for comparison. LDA scores ranged from −4.8 to 4.8. Cladograms of AN1, AN1 subanalysis, and AN2 are shown in Figure 3e–h, illustrating taxa grouping representation for all BC subgroups.

**Figure 4:**
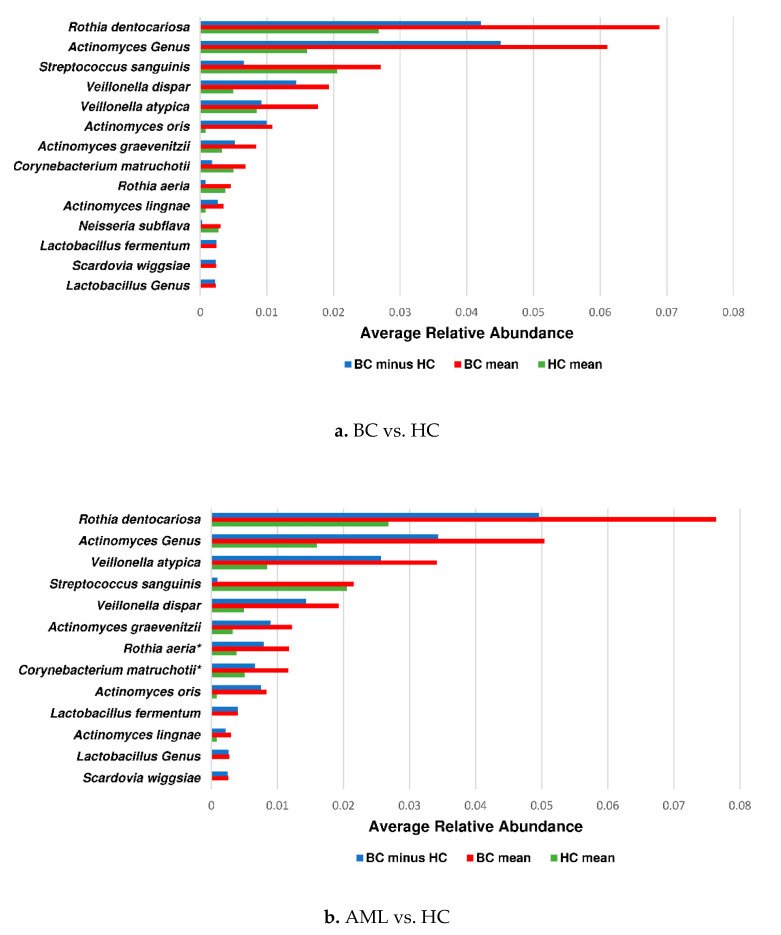
Differences in cumulative relative abundance between BC/AML pts and HC of select LEfSe identified distinctive species and genera. (**a**,**b**) Cumulative PST relative abundance data were used for bar chart representation for species and genera found significant by LEfSe, based on cumulative relative abundance data. * Nonsignificant distinguishing features per post-LEfSe Mann–Whitney test are marked by an asterisk (*p* ≥ 0.05). Mean represents cumulative PST mean.

We cumulated PST abundance data for the LEFSe features found significant in PST cumulative data to determine total “oral microbiome” relative abundances. Cumulative relative abundance data of LEFSe significant taxa for cumulative PST sample site data in AN1 and AN2 are illustrated in Figure 4a,b (AN1, AN2).

Furthermore, *Rothia* spp. *and Actinomyces* spp. accounted for large differences in cumulative relative abundance between the BC and HC groups in the PST sample site combination (Figure 4). A significant increase in relative abundance per oral sites P, S, and T in the BC group and the AML subgroup vs. the HC group was consistently observed for the *Actinomyces* genus (Figure S1a (AN1) and Figure S1b (AN2)).

No LEfSe-identified species were found to be at a lower relative abundance in the BC group compared to the HC group (AN1). Higher relative abundance was observed for *Rothia dentocariosa*, *Actinomyces* genus, and *Streptococcus sanguinis* (*p* < 0.05; Figure 4a). The AML subgroup in AN2 included lower relative abundance for the species *Neisseria subflava* and higher abundance differences for *Rothia dentocariosa* and *Actinomyces* genus (*p* < 0.05; Figure 4b).

**Figure 5:**
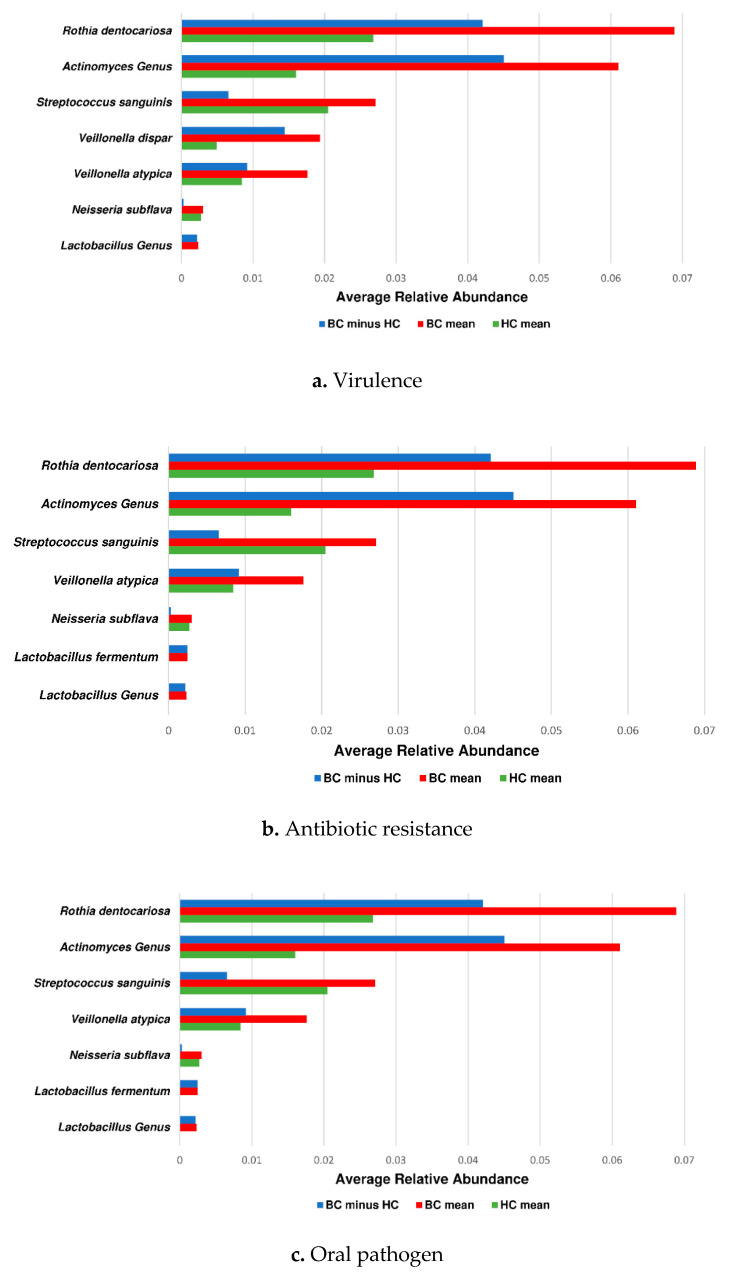
Average relative abundance of healthy controls compared to blood cancer patients for taxa associated with pathogenicity. Cumulative mean relative abundance data plots are shown for species/genera determined to be significant by LEfSe and associated with (**a**) at least 90% identity with virulence genes and (**b**) 100% identity with antibiotic resistance using the PATRIC_v3.6.7_ online tool. (**c**) Species/genera determined to represent oral pathogens by completing a manual search using PubMed and Google. These taxa were distinguishing features that were also found significant per post-LEfSe Mann–Whitney U-Test in the cumulative mean relative abundance BC vs. HC comparison. Mean represents cumulative PST mean.

Identification of significant differential features that may be involved in the changing microbial composition of BC pts was compared to HC warranted investigation of taxonomic profiles that may be related to pathogenicity concepts. Online program PATRIC_v3.6.7_ showed that both *Rothia dentocariosa* and *Actinomyces genus*, differentiating the BC group from the HC group, had over 90% identity with virulent factor genes and 100% identity with antibiotic resistance genes. In total, seven taxa had genes at least 90% identical to virulent factor genes, seven taxa had genes 100% identical to antibiotic-resistant genes, and eleven taxa were identified as opportunistic pathogens (Table 3). PST-cumulative RA charts show the species/genus abundance found significant per LEfSe analysis in the comparison of the BC group vs. the HC group, regarding pathogenicity concepts (Figure 5).

## 4. Discussion 

This is the largest study that has compared oral microbiome profiles of a variety of hematological cancers with those of healthy individuals by using oral samples, including saliva samples and buccal mucosa, superficial supragingival plaque, and tongue swabs, in a multivariate analysis. In agreement with Hu et al. [10], among the most prevalent genera in the oral cavities of our healthy control subjects were *Prevotella*, *Neisseria*, *Streptococcus*, *Haemophilus*, and *Fusobacterium.* In blood cancer patients, we also noted a significant decrease in *Haemophilus parainfluenzae*, a distinctive feature of healthy controls per LEfSe analysis (Figure 3). This species includes strains that are strong biofilm producers that are able to protect mucosal surfaces [17,22,23].

There were significant differences in the oral microbiome profile of hematological cancer patients (BC pts) regarding both *alpha* and *beta*-diversity when compared to HCs. 

LEfSe analysis indicated there were 13 species and 2 genera differentiating BC pts from HCs. The most prominent of the differential features were *Actinomyces* genus, *Rothia dentocariosa*, and *Veillonella dispar*. The *Actinomyces* genus was present regardless of antibiotic use among the BC group as a differential feature, rejecting the possibility that antibiotic use alone accounts for the change in the microbial composition of BC pts compared to HCs. Furthermore, we identified 17 species and 2 genera differentiating AML pts from HCs. *Rothia dentocariosa* and *Actinomyces* were the most prevalent differentiating feature (log LDA below −4.0) for both AN1 and AN2 comparisons. Distinct clustering was achieved between the BC group/AML subgroup and the HC group in both AN1 and AN2 comparisons (Figure 2). As anticipated, AB treatment of AML patients moved the clustering of the oral samples further apart from the HC group. *Actinomyces* genus was the largest differential feature for the BC group/AML subgroup compared to healthy controls (Figure 3e–h).

In addition, relative abundance data comparison showed a significant increase of *Rothia dentocariosa* and *Actinomyces* genus in hematological cancer patients compared to healthy control subjects. The abundance of these species increased particularly in plaque and tongue samples (Figure S1). For the PST site combination with cumulative relative abundance, the increase was observed for AML (10/11 pts above HC mean), LYM (7/8 pts above HC mean), MM (4/4 pts above HC mean), ALL (3/3 pts above HC mean), but not for MDS (1 pt). Additionally, CML (1 pt) and MF (1 pt) showed an increase of *Actinomyces* but a decrease in *Rothia dentocariosa*. This overall increase in *Actinomyces* spp. in the BC group was independent of strong antibiotic treatment effects (since the mean relative abundances differed similarly with the HC group) and was most likely not significantly associated with secondary variables such as gender/ethnicity. For *Actinomyces* (per genus probe), the PST cumulated mean relative abundance percentage was 0.016 for the HC group, 0.066 for the non-AB BC group, and 0.056 for the AB BC group (*p* < 0.01; BC groups vs. HCs, Mann–Whitney U-test).

*Actinomyces* genus corresponds to filamentous, nonspore-forming, Gram-positive bacilli, consisting of mostly facultative anaerobic species, including opportunistic pathogens (e.g., strictly anaerobe *Actinomyces israelli*, facultative anaerobe *A. gerencseriae*) most well-known for causing oral, thoracic, and intra-abdominal abscesses on rare occasions [24]. In addition, *Actinomyces* infection, known as actinomycosis, was previously reported to mimic malignancy symptoms, in particular lymphoma [25,26], whereas *Rothia dentocariosa* was historically confounded with *Actinomyces* spp. in selective growth conditions [27]. Ames et al. have also described an increase in the species *Rothia dentocariosa* in the oral cavity of cancer patients after allogeneic transplants, although overall microbial composition was consistent and cancer types were not described [16]. These results support the notion that in the presence of hematological cancer, there may be host factors capable of favoring lymphoma development systemically as well as *Actinomyces* colonization in the oral cavity. Our findings, therefore, raise the question as to whether *Actinomyces* spp. interact with the host in a manner that, in fact, would contribute to lymphoma (or other hematological cancer) progression or if BC contributes to an overall dysbiosis of the oral microbiome, leading to the progression of cancer. As multiple studies have suggested, it is likely that dysbiosis contributes to the development of cancer [28,29,30]. Indeed, an oral transfer to gut of the *Actinomyces* species could potentially activate inflammatory processes, as suggested by the work of Chua et al., who showed that increased abundance in phylum Actinobacteria in the gut was associated with increased immune activation in ALL survivors experiencing chronic inflammation [12]. In this respect, *Actinomyces* spp., normally commensals, were previously shown to invade damaged mucosa [31], possibly in the context of reduced mucosal protection by certain *Haemophilus parainfluenza* strains [17,22,23], although an acute infection may not occur due to host immune response. In addition, Jian et al. have recently shown that an increase in nitrogen-recycling bacteria in the gut, such as *Klebsiella* and *Streptococcus*, contributes to the progression of multiple myeloma, and the enrichment is likely due to an increase of urea nitrogen during the progression of the disease [32]. This warrants more investigation into whether the transfer of oral species to the gut could influence the development or progression of cancer.

Finally, using PATRIC_v3.6.7_ for the analysis of the pathogenicity concepts “antibiotic resistance”, “oral pathogen”, and “virulence”, based on LEfSe distinct oral microbiome profiles using cumulative PST data, we found that these concepts were significantly enriched in the blood cancer group (Table 3, Figure 5). The determination of potential for pathogenicity, based on oral microbiome profiles using PATRIC_v3.6.7_, showed that 7/17 species had at least 90% identity in their association with virulence (Figure 5a), and 7/17 species had 100% identity in their association with antibiotic resistance (Figure 5b). Both genera (*Actinomyces* and *Lactobacillus*) matched with at least 90% identity regarding their association with virulent genes and 100% identity with antibiotic-resistant genes. A total of 11 species was identified as opportunistic pathogens (Figure 5c), confirming their relevance to pathogenicity enrichment in the BC group.

Our findings are nevertheless subject to limitations. Even though we found overall commonalities of biological significance, the limited sample size for most cancer subgroups did not allow the identification of distinctive subgroups features with confidence. In addition, next-generation sequencing identifies live and dead bacteria so that it is unclear the extent to which *Actinomyces* species detected in saliva samples and tongue swabs are active. Obtaining metatranscriptomics and full metagenome data will probably shed more light on the determination of functionally enriched bacterial activities.

In conclusion, we note significant differences in oral microbiome *alpha* and *beta* diversities when comparing hematological cancer patients to healthy controls. More research is necessary to better understand how the oral microbiome interacts with its microenvironment and the host immune system to elucidate possible mechanistic pathways potentially favoring cancer development. Moreover, it remains to be determined whether the initiation of specific systemic oral microbiome-associated molecular interactions could serve as a biomarker for diagnosis or response to treatment of hematological cancers.

## Figures and Tables

**Table 1:** Demographics, clinical characteristics, and sample site combinations.

Variable			BC		HC			AML
			(N = 39)		(N = 27)			(N = 16)
Age yrs (mean (SD)) *		52.2 (15.08)		53.2 (14.07)			53.2 (14.97)	
Age range yrs			25–76		24–84			27–76
Gender (M/F) ^&^			15/24		7/19			9/7
Ethnicity ^$^								
	*Caucasian*		24		24			10
	*Black*		15		2			6
AB treatment		22		0			10	
Sample site combinations								
BPST (n = 132)				13		20		
BST (n = 24)			4		3			2
PST (n = 57)			16		3			7
ST (n = 12)			6		1			3
Total # of samples			n = 124		n = 100			n = 49

Blood cancers (BC, N = 39, n = 124) of the patient cohort included acute lymphoblastic leukemia (N = 3), acute myelogenous leukemia (AML; N = 16), chronic myelogenous leukemia (CML, N = 1), lymphoma (N = 10), myelodysplastic syndrome (N = 2), myelofibrosis (N = 2), and multiple myeloma (N = 5). Patients with AML represented the largest hematological cancer subgroup. BC pts may have been treated with antibiotics (AB) within two weeks prior to sampling, whereas none of the healthy control subjects were (HC; N = 27, n = 100). Oral microbiome sample sites were buccal mucosa (B), superficial supragingival plaque (P), saliva (S), and tongue (T). Statistics BC vs. HC group; * *p* = 0.9124 per Mann–Whitney U-test; ^&^ *p* = 0.3355 per chi-squared test; ^$^ *p* = 0.0046 per chi-squared test; pts is patients; M/F is gender male/female; # is number; SD is standard deviation, yrs is years.

**Table 2:** PERMANOVA analysis results for patients with hematological cancers or AML compared to healthy control subjects.

	PERMANOVA *p*-Value ^a,^*		
AN1 ^b^	Group ^a,d^	Site ^a,e^	AB-DIA ^a,f^
**BPST ^c^**	0.0008	0.0023	0.0001
HC vs. BC-No AB ^d^	0.0004	0.0003	0.1113
HC vs. BC-AB ^e^	0.0036	0.6155	0.0001
**PST ^f^**	0.0002	0.00001	0.00001
HC vs. BC-No AB ^g^	0.0001	0.0001	0.1544
HC vs. BC-AB ^h^	0.0032	0.0182	0.0002
HC vs. BC-AML, LYM, MM ^i^	0.0001	0.0001	0.1401
**BST ^j^**	0.0015	0.0056	0.0171
HC vs. BC-No AB ^k^	0.005	0.0031	0.2208
HC vs. BC-AB ^l^	0.0044	0.5188	0.0004
**ST ^m^**	0.0043	0.9524	0.0747
HC vs. BC-No AB ^n^	0.0107	0.0579	0.1245
HC vs. BC-AB ^o^	0.0017	0.3894	0.0083
**BPST cumulative ^p^**	0.1978	ND	0.0023
HC vs. BC-No AB ^q^	0.0909	ND	0.2512
HC vs. BC-AB ^r^	0.4825	ND	0.0012
**PST cumulative ^s^**	0.0857	ND	0.0028
HC vs. BC-No AB ^t^	0.04	ND	0.2687
HC vs. BC-AB ^u^	0.2548	ND	0.0026
HC vs. BC- AML, LYM, MM ^v^	0.0039	ND	0.2074
**ST cumulative ^w^**	0.0666	ND	0.1244
HC vs. BC-No AB ^x^	0.528	ND	0.0136
HC vs. BC-AB ^y^	0.0557	ND	0.2245
**AN2 ^z^**	**Group ^c^**	**Site ^d^**	**AB-DIA ^e^**
**HC vs. AML ^a,a^**	0.0002	0.0002	0.1087
**HC vs. NAML ^a,b^**	0.0001	ND	ND
**HC vs. YAML ^a,c^**	0.0001	ND	ND

^a^ PERMANOVA Monte-Carlo corrected *p*-values (*p* < 0.05) using PRIMER_v7_ software for multivariate permutational analysis of variance (PERMANOVA) show the microbial composition of the HC and BC groups are not equal by using a reducing model with unrestricted permutations of raw data (one factor) or permutation of residuals under a reduced model (2–3 factors), 9999 permutations, and type III partial sum of squares on two analyses for possible site combinations of buccal mucosa (B), superficial supragingival plaque (P), tongue (T) swabs, and saliva (S) samples. ^b^ Analyses 1 (AN1), where patients with blood cancer (BC) were compared to healthy control subjects (HC), with a subanalysis comparing HC with BC pts that did not receive or received antibiotics (AB). AN1 subcohort comparisons are as follows: ^c^ BPST: 20 HC/80 samples; 13 BC pts/52 samples; ^d^ BPST subanalysis of 20 HC/80 samples vs. 8 BC pts/32 samples that did not receive antibiotics (AB); ^e^ BPST subanalysis of 20 HC/80 samples vs. 5 BC pts/20 samples that did receive AB; ^f^ PST: 23 HC/69 samples; 29 BC pts/87 samples; ^g^ PST subanalysis of 23 HC/69 samples vs. 9 BC pts/45 samples that did not receive AB; ^h^ PST subanalysis of 23 HC/69 samples vs. 14 BC pts/42 samples that did receive AB; ^i^ PST subanalysis of 23 HC/69 samples vs. 23 BC pts/69 samples of BC type AML, LYM, and MM; ^j^ BST: 24 HC/72 samples; 17 BC pts/51 samples; ^k^ BST subanalysis of 24 HC/72 samples vs. 10 BC pts/30 samples that did not receive AB; ^l^ BST subanalysis of 24 HC/72 samples vs. 7 BC pts/21 samples that did receive AB; ^m^ ST: 26 HC/52 samples and 39 BC pts/78 samples; ^n^ ST subanalysis of 26 HC/52 samples vs. 17 BC pts/34 samples that did not receive AB; ^o^ ST subanalysis of 26 HC/52 samples vs. 22 BC pts/44 samples that did receive AB; ^p^ BPST cumulative: 20 HC/20 cumulative samples; 13 BC pts/13 cumulative samples; ^q^ BPST subanalysis of 20 HC/20 cumulative samples vs. 8 BC pts/8 cumulative samples; ^r^ BPST subanalysis of 20 HC/20 cumulative samples vs. 5 BC pts/5 cumulative samples; ^s^ PST: 23 HC/23 cumulative samples; 29 BC/29 cumulative samples; ^t^ PST subanalysis of 23 HC/23 cumulative samples vs. 15 BC/15 cumulative samples; ^u^ PST subanalysis of 23 HC/23 cumulative samples vs. 14 BC/14 cumulative samples; ^v^ PST subanalysis of 23 HC/23 cumulative samples vs. 23 BC pts/23 samples of BC type AML, LYM, and MM; ^w^ ST: 26 HC/26 samples; 39 BC pts/39 samples; ^x^ ST subanalysis of 26 HC/26 cumulative samples vs. 17 BC/17 cumulative samples; ^y^ ST subanalysis of 26 HC/26 cumulative samples vs. 22 BC/22 cumulative samples; ^z^ Analyses 2 (AN2) where acute myelogenous leukemia (AML) patients were compared to healthy control subjects; AN2 subcohort comparisons are as follows: ^a,a^ PST subcohort consisting of all AML pts regardless of AB use (23 HC/69 samples; 11 AML/33 samples); ^a,b^ PST subcohort where HC were compared to AML patients that had not received antibiotics (NAML; 23 HC/69 samples; 6 NAML/18 samples); ^a,c^ PST subcohort where the HC group was compared to AML patients who received antibiotics (YAML; 23 HC/69 samples; 6 YAML/18 samples); The following describe the PERMANOVA design: ^a,d^ fixed variable “Group” (BC, HC); ^a,e^ fixed variable “Site” (B, P, S, T); ^a,f^ random variable “AB-DIA” corresponding to “Antibiotics” (Y, N) combined with “Diagnosis” (HC, ALL, AML, CML, LYM, MDS, MF, MM) and nested into “Group” and “Site”. For example, no antibiotic use and healthy control subjects correspond to NHC. * Significance level was set at *alpha* = 0.05; ND is not determined.

**Table 3:** PATRIC_v3.6.7_ virulence, antibiotic resistance, and oral pathogen determination of LEfSe-identified blood cancer features.

Species/Genus ^a^	Virulence ^b^	Source ^c^	PMID ^d^	ABR ^e^	Source ^f^	Accession IDs ^g^	Oral Pathogen ^h^	PMID ^i^
***Actinomyces***	Yes	PATRIC_VF; Victors	10456927; 14600232; 1500984	Yes	NDARO; CARD	WP_000804064.1; WP_002586627.1; WP_063856422.1; WP_000691759.1; WP_000027050.1	Yes	30225251 23673380
***Actinomyces graevenitzii***	No			No			ND	
***Actinomyces lingnae***	ND			ND			ND	
***Actinomyces oris***	No			No			ND	
***Corynebacterium matruchotii***	No			No			ND	
***Lactobacillus***	Yes	VFDB; Victors; PATRIC_VF	12207705; 15937179; 14569030; 8063392; 19818015	Yes	ARDB; NDARO; CARD;	WP_002352254.1; WP_001038795.1; WP_002328813.1; ABI81768.1; WP_000027050.1	Yes	25758458
***Lactobacillus fermentum***	No			Yes	NDARO; CARD	AAF86220.1; WP_002328813.1; WP_011100845.1	Yes	19088910
***Neisseria subflava***	Yes	Victors; VFDB;	11062540; 19481311; 16988225; 19050914; 18680551	Yes	NDARO; CARD	WP_063856397.1; CAD09800.1; WP_000027057.1; AAL59753.1; WP_000480968.1	Yes	12324342
***Rothia aeria***	No			No			Yes	24951810
***Rothia dentocariosa***	Yes	Victors	20485570; 9383163;	Yes	NDARO; CARD	CAJ67339.1; WP_000691727.1	Yes	27303245
***Scardovia inopinata***	No					No	Yes	32401932
***Scardovia wiggsiae***	No			No			Yes	29104444
***Streptococcus sanguinis***	Yes	Victors; PATRIC_VF	8820650; 12207705; 15731074; 10768978; 10998175;	Yes	NDARO; CARD	CAA45935.1; WP_000420317.1; WP_000420313.1; WP_000417519.1; WP_000691736.1	Yes	32082276
***Veillonella atypica***	Yes	Victors	11062540	Yes	NDARO; CARD	WP_000018329.1; CAA45935.1; WP_001038790.1; WP_000196697.1;	Yes	28473967
***Veillonella dispar***	Yes	Victors	11062540; 12207705	No			Yes	28473967

^a^ Species/genus based on LEfSe results of the BC group (HC distinguishing taxa not shown). ^b^ Virulence associated with at least a 90% identity with one or more virulent factor genes using the search term “virulence” under “special features” in the PATRIC_v3.6.7_ online database. ^c,f^ Source database for which identity was determined. ^d^ PATRIC_v3.6.7_ PubMed identification numbers (PMIDs) associated with the concept “virulence”. ^e^ Antibiotic resistance (ABR) association with a 100% identity, with one or more antibiotic-resistant genes, using the search term “antibiotic resistance” under “special features” in PATRIC_v3.6.7_. ^g^ Accession identification (ID) codes of antibiotic-resistant geness matching 100% identity. ^h^ Manual search results for determining the association with the concept “oral pathogenesis” of species/genera; ^i^ PMIDs associated with the concept “oral pathogenesis” of species/genera. CARD stands for Comprehensive Antibiotic Resistance Database (https://card.mcmaster.ca); NDARO stands for National Database of Antibiotic-Resistant Organisms (https://www.ncbi.nlm.nih.gov/pathogens/antimicrobial-resistance). ND is not determined.

## Supplementary Materials

The following are available online at https://www.mdpi.com/2077-0383/9/12/4068/s1, Table S1: Species and genera identification for the BC and HC groups; Figure S1: Average number of taxa per subject and sample site combinations; Supplementary Data File: Raw taxa abundance data of BC and HC oral samples.
