# Supplementary material for: Oral Microbiome Signatures in Hematological Cancers Reveal Predominance of Actinomyces and Rothia Species
Source: J Clin Med. 2020 Dec 17;9(12):4068. doi: 10.3390/jcm9124068 (PMC7767039; doi:10.3390/jcm9124068)
Supplement: Supplementary file 1 [file jcm-09-04068-s001.pdf]

Table S1. Species and genera identification for hematological cancer patients and healthy control subjects.

	**Probes not detected**[a]			**Probes detected**[b]		
	Genera[c]	Species[d]	Total[e]	Genera[c]	Species[d]	Total[e]
**BPST**[f]	16	134	150	62	400	462
**PST**[g]	18	140	158	60	394	454
**BST**[h]	16	140	156	62	394	456
**ST**[i]	19	135	154	59	399	458
**BPST cumulative**[j]	16	134	150	62	400	462
**PST cumulative**[k]	18	139	157	60	395	455
**ST cumulative**[l]	19	144	163	59	390	449

Species/genera identification of hematological cancer patients (BCs, n=39) and healthy control subjects (HC, n=27) considering 534 possible species and 78 genera (612 total taxa) through species and genus probes with processed duplicates from oral samples of buccal mucosa (B), superficial supragingival plaque (P), tongue swabs (T), and saliva samples (S). [a]Number of species/genera probes not detected or [b]number of species/genera probes detected using Human Oral Microbe Identification using *Next Generation Sequencing* (HOMI*NGS*) 16S rRNA gene (V3-V4 region). [c] Number of genera not detected or detected. [d] Number of species not detected or detected. [e]Total number of probes (total not detected or total detected). [f]BPST sub-cohort (20 HCs/80 samples; 13 BCs/52 samples). [g]PST sub-cohort (23 HCs/69 samples; 29 BCs/87 samples). [h]BST sub-cohort (24 HCs/72 samples; 17 BCs/51 samples). [i]ST sub-cohort (27 HCs/54 samples; 39 BCs/78 samples). [j]BPST cumulative sub-cohort (20 HCs/20 cumulated samples; 13 BCs/13 cumulated samples). [k]PST cumulative sub-cohort (23 HCs/23 cumulated samples; 29 BCs/29 cumulated samples). [l]ST cumulative sub-cohort (27 HCs/27 cumulated samples; 39 BCs/39 cumulated samples.

Table S2. Average number of species and genera identified per subject for different sample site combinations analyzed in patients with blood cancer *versus* healthy controls.

**Average number of taxa detected**[a]		
**AN1**[b]	**HC**[c]	**BCs**[d]
BPST[e]	143.5	95.56
PST[f]	145.26	99.61
BST[g]	148.43	99.73
ST[h]	153.02	106.13
BPST cumulative[i]	221.6	150.62
PST cumulative[j]	172.78	118.48
ST cumulative[k]	187.74	136.85
**AN2**[l]	**HC**[c]	**AML**[m]
PST HC *vs.* AML[n]	145.26	108.06
PST HC *vs.* NAML[o]	145.26	118.83
PST HC *vs.* YAML[p]	145.26	95.13

[a]Average number of species/genera detected for different sample site combinations analyzed. [b]Analyses 1 (AN1) where [c]healthy controls (HC) were compared to patients with [d]blood cancers (BCs). [e]BPST sub-cohort (20 HCs/80 samples; 13 BCs/52 samples). [f]PST sub-cohort (23 HCs/69 samples; 29 BCs/87 samples). [g]BST sub-cohort (24 HCs/72 samples; 17 BCs/51 samples). [h]ST sub-cohort (27 HCs/54 samples; 39 BCs/78 samples). [i]BPST cumulative sub-cohort (20 HCs/20 cumulated samples; 13 BCs/13 cumulated samples). [j]PST cumulative sub-cohort (23 HCs/23 cumulated samples; 29 BCs/29 cumulated samples). [k]ST cumulative sub-cohort (27 HCs/27 samples; 39 BCs/39 samples). [l]Analyses 2 (AN2) where [m]Acute Myelogenous Leukemia (AML) patients were compared to HCs. [n]PST sub-cohort where HCs were compared to all AML patients (23 HCs/69 samples; 11 AMLs/33 samples). [o]PST sub-cohort where HCs were compared to AML patients that had not received antibiotics (NAML) (23 HCs/69 samples; 6 NAML/18 samples). [p]PST sub-cohort where HCs were compared to AML patients that had received antibiotics (YAML) (23 HCs/69 samples; 6 YAML/18 samples).

a. BCs group *vs.* HC group per oral site

Plaque

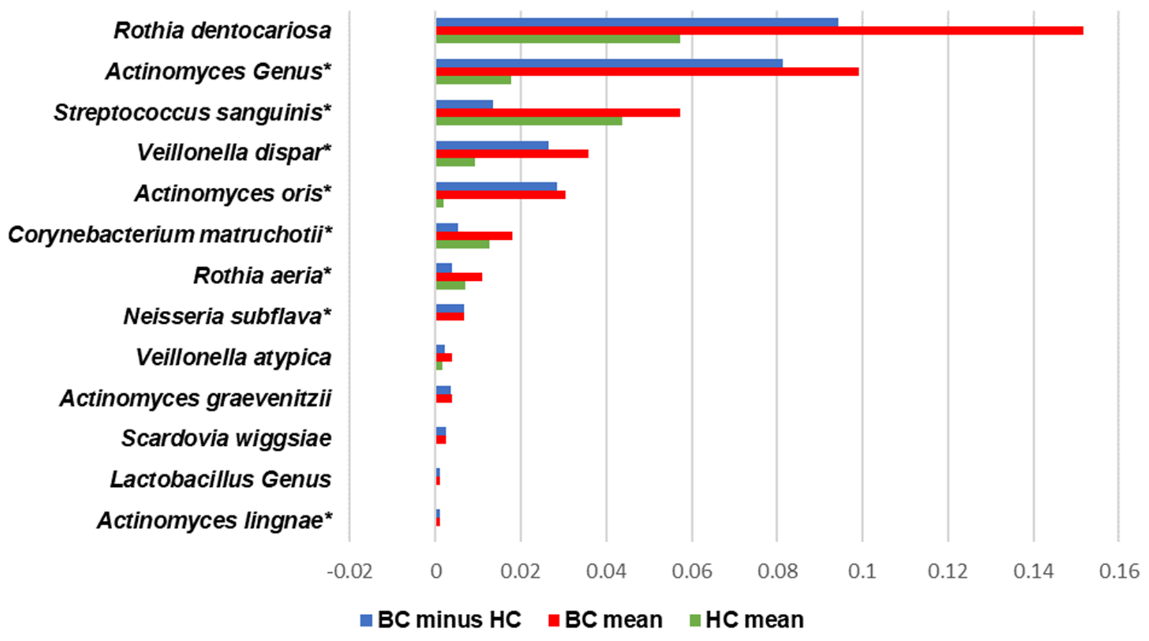

Saliva

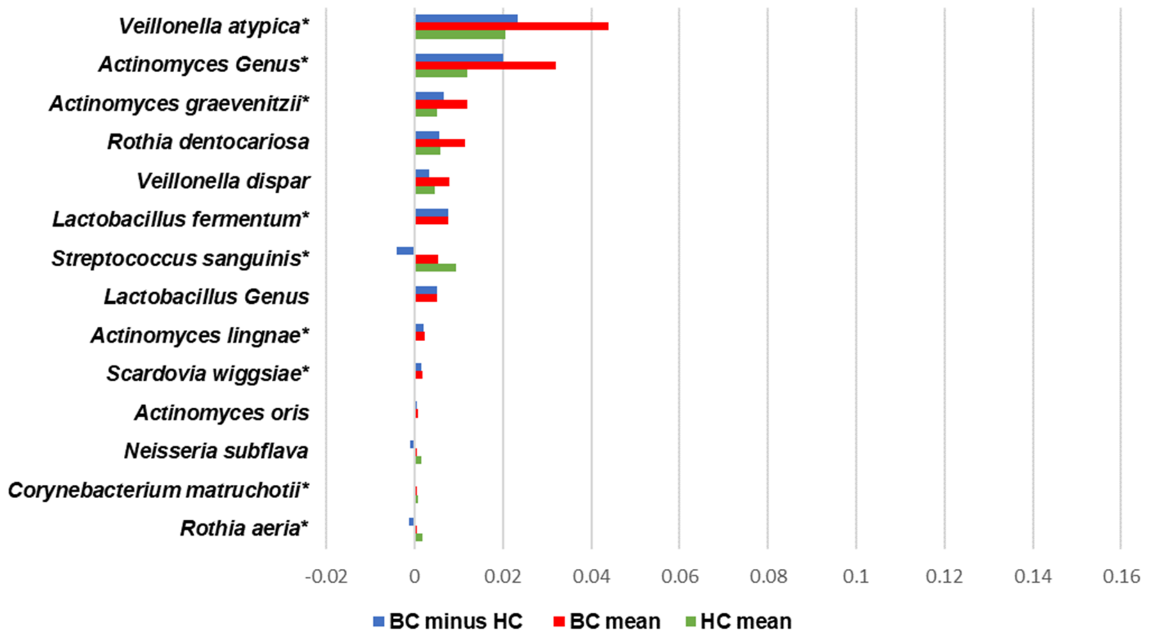

Tongue

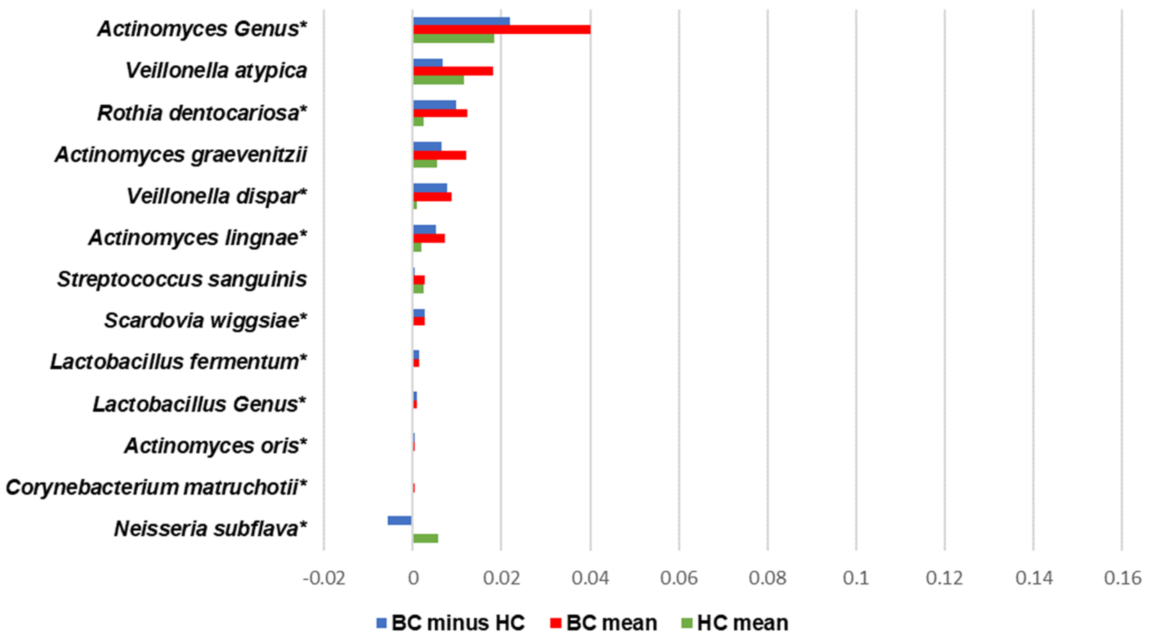

b. AML subgroup *vs.* HC group per oral site

Plaque

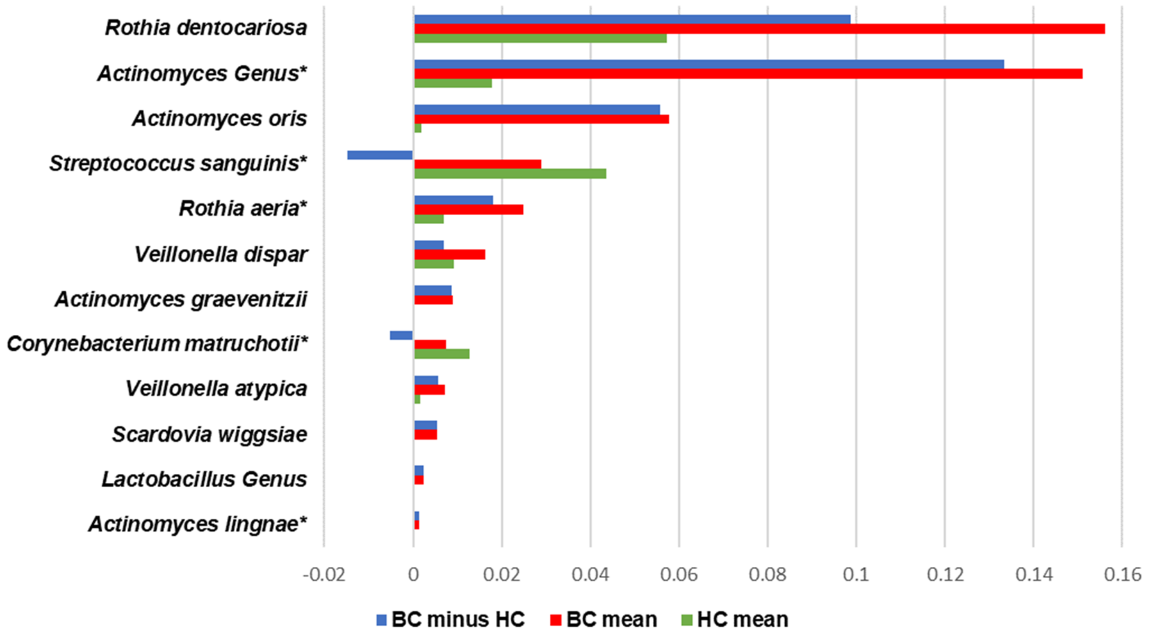

Saliva

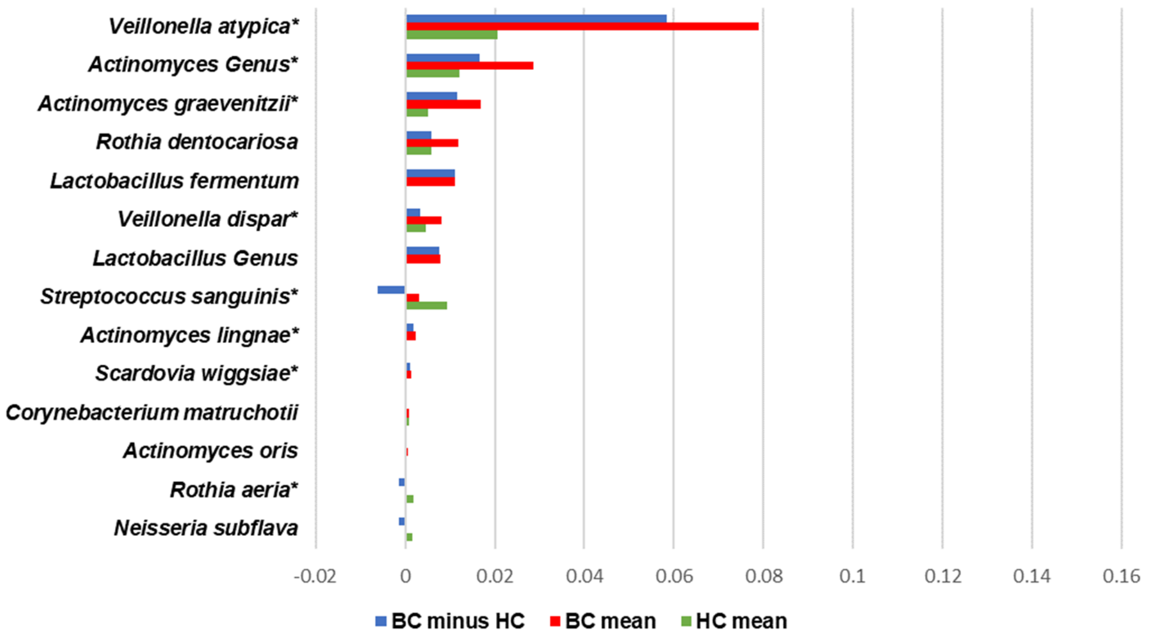

Tongue

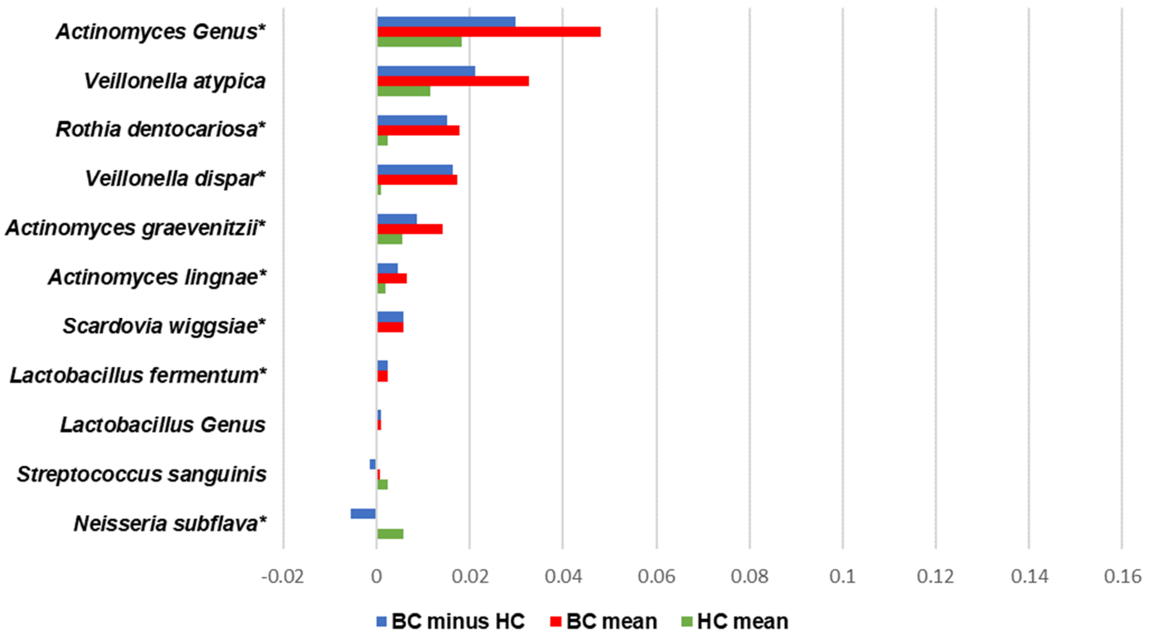

Figure S1. Differences in non-cumulative relative abundance between BC group and AML subgroup and HC group of LEfSe-identified distinctive species and genera. (**a, b**) Non-cumulated relative PST data for species and genera found significant by LEfSe as distinctive features for the hematological cancer (BCs) group (**a**) or acute myeloid leukemia (AML) subgroup (**b**) compared to healthy controls (HC group). *Significant distinguishing features per post-LEfSe Mann-Whitney test are marked by an asterisk ($p<0.05$). Negative values are shown with a hyphen (-); positive values have no indicator.
